# Patientensicherheit bei differenzierter (innerklinischer) Schockraumaktivierung für Schwerverletzte

**DOI:** 10.1007/s00113-022-01279-5

**Published:** 2023-01-09

**Authors:** S. Hagel, K. R. Liedtke, S. Bax, S. Wailke, T. Klüter, P. Behrendt, G. M. Franke, A. Seekamp, P. Langguth, A. Balandin, M. Grünewald, D. Schunk

**Affiliations:** 1 Klinik für Unfallchirurgie und Orthopädie, Universitätsklinikum Schleswig-Holstein, Campus Kiel, Kiel, Deutschland; 2 Klinik für Anästhesiologie, Universitätsklinikum Schleswig-Holstein, Campus Kiel, Kiel, Deutschland; 3 Zentrale Einrichtung Interdisziplinäre Notaufnahme, Universitätsklinikum Schleswig-Holstein, Campus Kiel, Kiel, Deutschland; 4 Klinik für Radiologie und Neuroradiologie, Universitätsklinikum Schleswig-Holstein, Campus Kiel, Kiel, Deutschland

**Keywords:** Polytrauma, Schockraumalarmierung, Notaufnahmeteam, Schockraumversorgung, Schwerstverletztenversorgung, Polytrauma, Resuscitation room activation, Emergency department team, Resuscitation room treatment, Seriously injured care

## Abstract

**Hintergrund und Fragestellung:**

Das Vorhalten einer Schockraumbereitschaft erfordert hohe personelle und instrumentelle Kosten. Das Ziel dieser Studie war es, die Versorgungsressourcen durch die Kategorisierung in einen A‑Schockraum und einen B‑Schockraum als modifiziertes Versorgungskonzept auf seine Sicherheit und Praktikabilität zu prüfen.

**Methodik:**

In einer prospektiven, monozentrischen Studie wurden traumatologische Schockräume im Zeitraum von Mai 2020 bis Januar 2021 anhand der S3 Leitlinien GoR A und B Kriterien und des ABCDE-Schemas in A‑Schockräume (SR-A) oder B‑Schockräume (SR-B) eingeteilt. Die Einteilung der Schockräume erfolgte nach telefonischer Anmeldung bei dem Oberarzt der Notaufnahme. Neben den Vitalparametern bei der Patientenaufnahme wurden die Hospitalisierungsdauer sowie die Mortalität erhoben. Die Gruppenvergleiche erfolgten mittels *t*-Test, Chi-Quadrat-Test oder Mann-Whitney-U-Test verwendet und ein *p* < 0,05 als signifikant definiert.

**Ergebnis:**

Im Studienzeitraum wurden 135 Schockräume erfasst. Davon waren 93 Schockraumanmeldungen dem SR-B- und 42 dem SR-A-Team zugeteilt worden. In der Zuweisung zeigte sich, dass bei SR-A-Trauma-Patienten in 80,5 % eine Notarztbegleitung erfolgte, während dies bei dem SR-B-Patienten bei 55,5 % lag. Der Glasgow Coma Scale (GCS) war bei den SR‑B Patienten signifikant höher. In der Notfallsonographie (eFAST) konnte in einem Fall der SR‑A und in vier Fällen des SR‑B ein traumaassoziierter pathologischer Befund festgestellt werden. Eine Übernahme auf die Intensivstation war in 26 % bei SR‑A und 3,2 % bei SR‑B notwendig. Neun SR‑A Patienten und ein SR‑B Patient verstarben innerhalb von 30 Tagen. Der Injury Severity Score (ISS) zeigte eine signifikant größere Verletzungsschwere bei den Patienten im SR‑A zu den Patienten im SR‑B (ISS 28,3 vs. 6,8).

**Schlussfolgerung:**

Die Ergebnisse dieser Studie zeigen, dass die innerklinische Zuordnung in einen A‑ und einen B‑Schockraum nach dem Polytraumameldebild in Verbindung mit dem gemeldeten ABCDE-Status sicher anzuwenden ist. Dabei werden die personellen Schockraumressourcen effizient eingesetzt, ohne dass es einen Hinweis auf eine gesteigerte Patientengefährdung durch diese Form der Zuteilung gibt.

**Graphic abstract:**

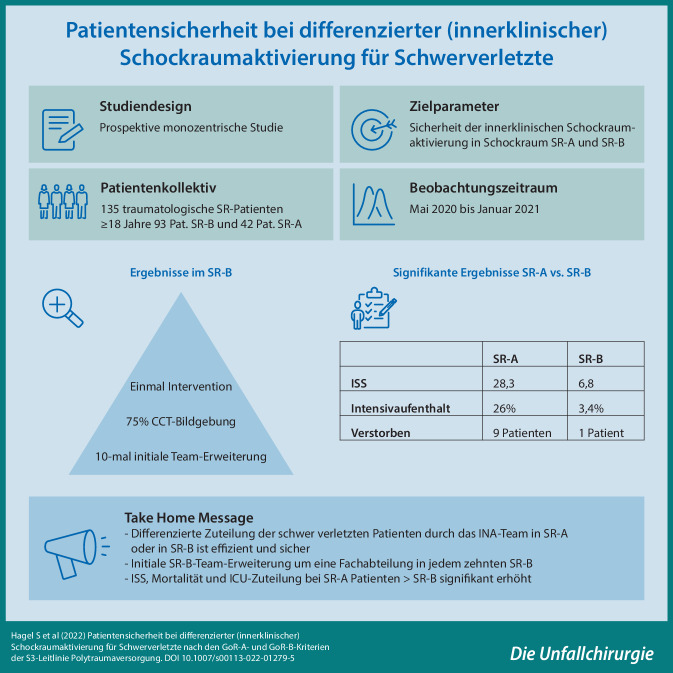

## Abstract (en)

**Background and objective:**

Providing trauma services demands high personnel resources and structural costs. The goal of this study was to show if the assignment of trauma patients to a defined A or B resuscitation room treatment as a modified management concept is safe and feasible.

**Material and methods:**

Between May 2020 and January 2021, all resuscitation room trauma patients were included in this single center prospective observational study. All patients admitted to the resuscitation room underwent a classification according to the German S3 guidelines grade of recommendation GoR A and GoR B in polytrauma and the status of the ABCDE sequence to an A and B resuscitation room. The classification was performed by the senior consultant on call via telephone after consultation and discussion of clinical findings.

**Results:**

All 135 resuscitation room patients were included in the study of whom 42 trauma patients were assigned to the A resuscitation room (A-SR) and 93 were assigned to the B resuscitation room (B-SR). The comparison of the two groups showed that patients in the A‑SR group are more likely to be accompanied by a prehospital emergency physician (80.5%) than patients in the B‑SR group (55.5%). Patients in the B‑SR group showed a significantly higher Glasgow coma scale (GCS). Using the eFAST emergency ultrasound protocol, 2.4% of the A‑SR and 4.3% of the B‑SR patients had trauma-associated pathologies, 26% of the A‑SR and only 3.2% of the B‑SR patients had to be admitted to the ICU, 21.4% of the A‑SR and 1% of the B‑SR patients died within 30 days after trauma. The injury severity scores (ISS) of the A‑SR patients were significantly higher than in the B‑SR group (ISS 28.3 vs. 6.8).

**Conclusion:**

The study confirmed that the assignment by the emergency department consultant according to the German S3 guideline in combination with the ABCDE sequence into resuscitation room A or B treatment is feasible, does not compromise the quality of care and is efficient in the use of the personnel resources.

Die hohe Zahl von 32.000 schockraumpflichtigen Patienten in Deutschland pro Jahr bedingt einen hohen Vorhalteaufwand in Kliniken, obwohl nicht alle Patienten, die die Schockraumkriterien erfüllen, auch schwerstverletzt sind. Eine modifizierte innerklinische Schockraumzuordnung nach dem Weißbuch Schwerverletztenversorgung der deutschen Gesellschaft für Unfallchirurgie (DGU) in einen Schockraum GoR A und GoR B, soll den Vorhalteaufwand reduzieren, ohne die Patientensicherheit und das Outcome zu beeinträchtigen.

## Einleitung

In Deutschland verletzten sich im Jahr 2018 ca. 32.000 Menschen bei Verkehrs‑, Sport- und Freizeit- sowie Arbeits- und häuslichen Unfällen so schwer, dass sie zur weiteren Versorgung über den Schockraum eines Traumazentrums vorgestellt wurden [1]. Die Behandlung von Schwerstverletzten stellt eine besondere Herausforderung dar und ist eine interdisziplinäre Aufgabe. Hierzu existieren international anerkannte evidenz- und konsensbasierte Empfehlungen. Die vollumfängliche Versorgung der schwer verletzten Patienten in einem Traumazentrum erfordert eine Vorhaltung von Schockraum‑, Intensiv‑, OP-, Diagnostik- und Personalkapazitäten nach Maßgaben des Weißbuches für die Schwerverletztenversorgung der Deutschen Gesellschaft für Unfallchirurgie (DGU).

Ziel der präklinischen und klinischen Zusammenarbeit ist eine sichere und effektive Versorgung schwer verletzter Notfallpatienten. Dabei spielen die lokalen Krankenhausstrukturen und die hohen Anforderungen an die Schockraumversorgung eine wesentliche Rolle, um der Patientenversorgung gerecht zu werden. Es gilt, insbesondere das Verletzungsmuster und -ausmaß des Patienten adäquat zu beurteilen und einerseits nicht zu unterschätzen (Untertriage) sowie andererseits nicht unnötig zu überschätzen (Übertriage) [2]. Eine Untertriage ist mit einer potenziellen Gefährdung für die Patienten assoziiert. Eine häufige Übertriage kann für die Krankenhäuser und das medizinische Personal neben einer zunehmenden physischen und psychischen auch eine ökonomische Belastung im Alltagsbetrieb darstellen und weitere Prozesse in der Krankenversorgung erheblich behindern. Mit dem Ziel, die personellen und instrumentellen Vorhaltekosten zu reduzieren und prozedurale Abläufe im Krankenhaus zu optimieren, wird an einigen Traumazentren ein modifiziertes Versorgungskonzept realisiert [3]. Hierzu wird in vielen überregionalen Traumazentren eine Trennung in ein Basisschockraumteam B oder das erweiterte Schockraumteam A vorgenommen.

In einigen Rettungsdienstbereichen und damit verbundenen Publikationen wird die Alarmierung des A- oder B‑Schockraums präklinisch durch den Notarzt im Rettungsdienst vor Ort entschieden und richtet sich nach der S3-Leitlinie Polytrauma/Schwerverletzten-Behandlung [4, 7].

Am Universitätsklinikum Schleswig-Holstein (UKSH), Campus Kiel wird seit Mai 2020 durch den verantwortlichen Oberarzt der interdisziplinären Notaufnahme (INA) mit Hilfe einer standardisierter Schockraumabfrage die Aktivierung und Zuteilung in einen A‑ oder B‑Schockraum ausgelöst. Dieses Vorgehen ist in den bisher vorliegenden Studien in Deutschland noch nicht beschrieben worden [4].

## Methodik

### Studiendesign

In einer prospektiven, monozentrischen Observationsstudie an einem universitären überregionalen Traumazentrum (ÜTZ) wurden die klinischen Daten von im Regelarbeitsbetrieb (Anwesenheit INA-Oberarzt 08:00–21:00 Uhr) über den Schockraum aufgenommenen Patienten in einem Zeitraum von 8 Monaten (Mai 2020 bis Januar 2021) erhoben. Hierfür ist das Ethikvotum der Medizinischen Fakultät der Christian-Albrechts-Universität zu Kiel im Vorweg eingeholt worden (AZ: D 493/20).

Im Vorfeld der Studie wurde in den Trauma- und INA-Qualitätszirkeln mit den beteiligten Schockraumfachdisziplinen die Kombination der S3-Leitlinien-Kriterien mit dem bekannten ABCDE-Schema sowie die fachliche Zusammensetzung für das Schockraum Basisteam SR‑B und das erweiterte Schockraumteam SR‑A definiert. Eine wesentliche zu lösende Frage hierbei war die Einbindung und Verantwortlichkeit der klinischen Akutmediziner in der eigenständigen Notaufnahme nach dem G‑BA-Beschluss von Mai 2018, da diese Rolle im Weißbuch der DGU für ein überregionales Traumazentrum (ÜRTZ) noch nicht definiert ist (Infobox, [5]).

Der Notaufnahme-Oberarzt als Akutmediziner wurde in allen Schockräumen (SR‑A und SR-B) als Schockraum-Leader eingesetzt und trug fachlich sowohl in der Zuordnung (SR‑A vs. SR-B) als auch in der Leitung des Schockraums die Hauptverantwortung. Somit sicherte er als konstanter Bestandteil in allen Schockräumen die akutmedizinische Fachlichkeit.

**Figure 1:**
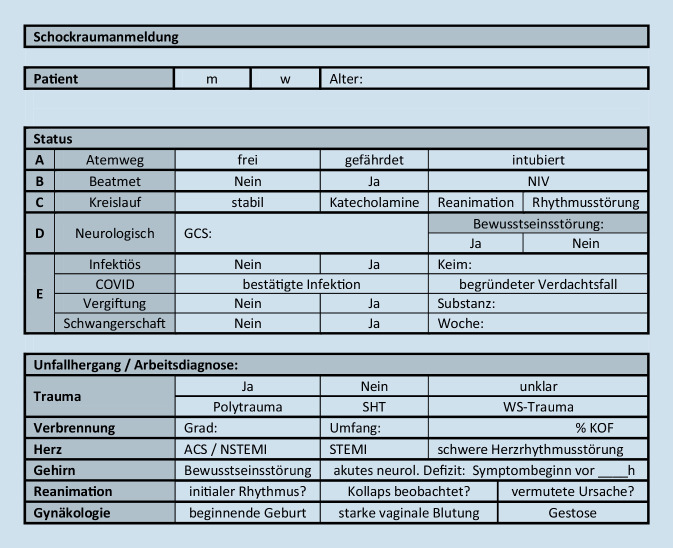

Die innerklinische Schockraumindikation und damit verbundene Ressourcenaufteilung in Schockraum A und B (Abb. 1) erfolgte anhand folgender Kriterien nach telefonischer Abfrage durch die Leitstelle bzw. durch den annehmenden Oberarzt am Schockraumtelefon bei direkter Alarmierung ohne Leistelle:

- **SR‑A**: offensichtlich schwere Verletzung und/oder Verletzung mit oder ohne Störung von Vitalparametern oder mindestens eine lebensbedrohliche Störung im ABCDE-Schema. Vergleichbar mit der GoR-A-Empfehlung der S3-Polytrauma-Leitlinie (Tab. 1),
- **SR-B: **potenziell schwer verletzter Patient aufgrund des Unfallmechanismus (GoR B; Tab. 1) und zum Zeitpunkt der Anmeldung mit stabilen Vitalparametern. Im ABCDE-Schema keine oder nur geringfügige Einschränkung ohne vitale Bedrohung des Patienten.

**Table 1:** 

GoR A – Polytrauma-Schockraum	GoR B – Polytrauma-Schockraum
Systolischer Blutdruck < 90 mm Hg nach Trauma (altersadaptiert bei Kindern)	Sturz aus über 3 m Höhe
Vorliegen von penetrierenden Verletzungen/Schussverletzungen der Rumpf-Hals-Region	*Verkehrsunfall (VU) mit:*
GCS < 9 nach Trauma	Frontalaufprall mit Intrusion von mehr als 50–75 cm
Atemstörungen/Intubationspflicht nach Trauma	Einer Geschwindigkeitsveränderung von Delta > 30 km/h
Frakturen von mehr als 2 proximalen Knochen	Fußgänger- /Zweiradkollision
Instabiler Thorax	Tod eines Insassen
Beckenfrakturen	Ejektion eines Insassen
Amputationsverletzung proximal der Hände/Füße	
Querschnittsverletzung	
Offene Schädelverletzungen	
Verbrennungen > 20 % KOF und Grad ≥ 2b	

Von den präklinischen Rettungsdienstteams, inklusive der luftgebundenen Standorte in und um Kiel (Radius 100 km), wurde über die integrierte Rettungsleitstelle (IRLS) eine Schockraumindikation nach dem mit den Kieler Kliniken konsentierten Leitstellenschema angemeldet (Abb. 1). Hierzu wurden dem diensthabenden Oberarzt der Notaufnahme telefonisch die Eckdaten wie Alter der verletzten Person, Geschlecht, Unfallzeitpunkt, das Verletzungsmuster, die Unfallart/-kinetik und das ABCDE-Schema mitgeteilt.

Anhand des Meldebildes und der mitgeteilten Informationen wurde die Entscheidung getroffen, welches Schockraumteam und ob ggf. weitere Ressourcen zu aktivieren sind. Es sind sowohl bei SR‑A als auch SR‑B alle potenziellen Schockraummitglieder über die Alarmierung und Kategorisierungsentscheidung informiert worden, um sicherzustellen, dass das gesamte Schockraumteam bei einer nichtmitgeteilten Zustandsverschlechterung des Patienten oder einer fehlerhaften Kommunikation ohne Zeitverzug nachalarmiert werden kann.

Ausschlusskriterien für die Teilnahme an dieser Studie waren eine Vorstellung außerhalb der Kernarbeitszeit (i.e. zwischen 21:00 und 08:00 Uhr) sowie Patienten, die kein Trauma erlitten hatten, jünger als 18 Jahre alt waren oder als Sekundärverlegung aus einem anderen Krankenhaus aufgenommen wurden.

Die Zuteilung zu den Schockräumen erfolgte rein über die Erfassung anhand der Abfragebogen. Es wurden alle bildgebenden Untersuchungen (CT, Röntgen, Sonographie (extended Focussed Assessment Sonography in Trauma, eFAST), MRT), die im direkten zeitlichen Zusammenhang mit dem Primary und Secondary Survey des Schockraummanagements erfolgten, erfasst.

### Outcome-Parameter

Es wurden das präklinische Meldebild nach GoR A oder B, inklusive des ABCDE-Status, erfasst, die Begleitung durch den Notarzt, die innerklinische Entscheidung, ob ein SR‑A oder B ausgelöst wurde, sowie die Teamstärke.

Darüber hinaus wurden die Glasgow-Coma-Scale (GCS), der Injury Severity Score (ISS), Blutdruck bei Eintreffen, das Ergebnis der Sonographie (eFAST) und im Schockraum erfolgte Interventionen erfasst. Die 30-Tage-Mortalität wurde für alle Schockraumpatienten dokumentiert.

### Statistische Methoden

Nichtkategoriale Studiendaten wurden mittels Kolmogorov-Smirnov-Tests auf Normalverteilung überprüft und mittels *t*-Test für ungepaarte Stichproben analysiert. Kategoriale Variablen wurden mittels Chi-Quadrat-Test analysiert. Ein Signifikanzniveau von *p* < 0,05 wurde festgesetzt. Eine vorherige Fallzahlberechnung erfolgt nicht. Die Datenauswertung erfolgte hierbei rein deskriptiv und vergleichend. Die Studie war ursprünglich von Ende Januar 2020 für ein Jahr geplant. Durch die sich anbahnende Coronapandemie und die Vorbereitungen auf die erste Welle wurde die Studie erst im Mai 2020 gestartet.

## Ergebnisse

### Patientenkollektiv

In dem Studienzeitraum wurden 256 Schockraumversorgungen durchgeführt, von diesen erfüllten 135 (53 %) die Einschlusskriterien für diese Studie. Die übrigen 121 Schockraumanmeldungen wurden aufgrund der definierten Ausschlusskriterien ausgeschlossen (s. oben).

Von den analysierten 135 Schockraumanmeldungen waren innerklinisch 42 (31,1 %) der SR-A- und 93 (68,9 %) der SR-B-Versorgung zugeordnet worden. Die Altersspanne der 135 Patienten betrug 19 bis 86 Jahre (Median: 53 Jahre) bei den SR-A-Patienten sowie 18 bis 95 Jahre (Median: 48 Jahre) bei den SR-B-Patienten. Weibliche Patienten hatten einen Anteil von 24 % (*n* = 10) der SR-A- und 30 % (*n* = 28) der SR-B-Patienten.

In 9 von 42 Fällen (21 %) der SR-A-Patienten war der Patient bereits endotracheal intubiert bzw. musste im Schockraum intubiert werden. Dahingegen benötigte keiner der SR-B-Patienten eine Intubation.

Von den SR-A-Patienten mussten 11 (26,2 %) und von den SR-B-Patienten 3 (3,2 %) unmittelbar auf einer Intensivstation weiterbehandelt werden.

### Präklinische Rettungsmittelzuweisung

**Figure 2:**
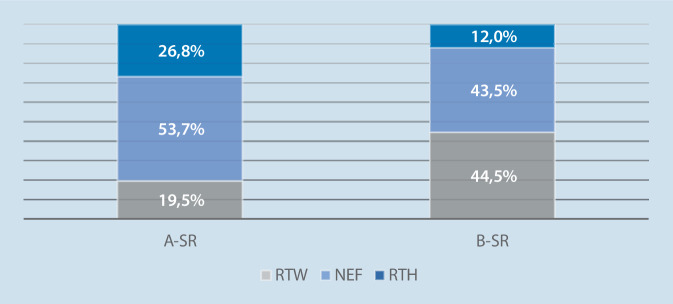

Die Schockraumanforderung wurde boden- und luftgebunden durch den Rettungsdienst über die IRLS angemeldet. Mit Notarztbegleitung wurden 80,5 % der SR-A- und 55,5 % der SR-B-Fälle im Schockraum vorgestellt (Abb. 2). Ob initial ein Notarzt vor Ort gewesen ist und nur der Transport ohne Arztbegleitung stattgefunden hat, konnte in dieser Arbeit nicht erhoben werden.

### Schockraumteamerweiterung

In 10 von 93 (10,75 %) SR‑B wurde bereits initial aufgrund des Unfallmechanismus, Verletzungsmusters oder einer nichtbedrohlichen (leichten) Störung im ABCDE-Schema eine weitere Fachdisziplin hinzugezogen. Die Allgemein- und Thoraxchirurgie wurde in 4 Fällen, die Neurochirurgie in 5 Fällen und die Mund-Kiefer-Gesichtschirurgie mit der Ophthalmologie in einem Fall gemeinsam parallel zum SR‑B alarmiert. In einem Fall erfolgte aufgrund einer präklinischen Intubation durch den begleitenden Notarzt noch vor Eintreffen des Patienten die Konversion von einer SR-B- in eine SR-A-Anmeldung. In keinem Fall war innerklinisch eine vollständige personelle Konversion des SR‑B in einen SR‑A notwendig.

Hingegen wurde in der Versorgung der SR-A-Patienten nur in 2 von 42 Fällen (4,8 %) eine weitere Fachdisziplin in Form der Inneren Medizin (*n* = 1) und Neurologie (*n* = 1) ergänzt.

### Erhobene Parameter

Der systolische Blutdruck und der GCS waren bei den SR-A Patienten signifikant niedriger, während die Herzfrquenz in diesem Patientenkollektiv zugleich signifikant erhöht war (Tab. 2). Der GCS war bei den SR-A-Patienten signifikant niedriger als bei den SR-B-Patienten.

**Table 2:** 

Parameter	SR‑A	SR‑B	*p*-Wert
RR_sys_	133 ± 32 mm Hg	143 ± 22 mm Hg	0,0094
Herzfrequenz	92 ± 32 min^−1^	85 ± 17 min^−1^	< 0,0001
GCS	11,1 ± 5,9	14,7 ± 5,1	< 0,0001

Wertebereich angegeben als Mittelwert mit Standardabweichung

**Figure 3:**
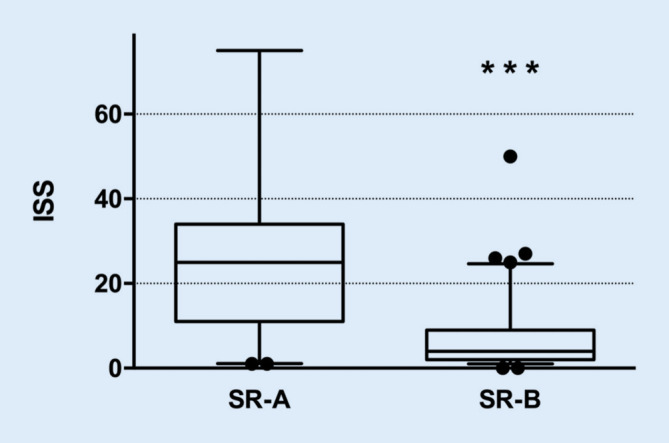

Eines der Ziele dieser Studie war es, die innerklinischen Parameter, anhand derer weniger schwer verletzte Patienten erkannt und mit einem reduzierten personellen Aufwand im Schockraum angenommen und behandelt werden können, hinsichtlich ihrer Praktikabilität und Sicherheit hin zu untersuchen. Die Schwere der Verletzung wurde in dieser Arbeit mit dem Injury Severity Score (ISS), welcher einfach anzuwenden und weltweit in Anwendung ist, quantifiziert. Die Auswertung belegt, dass die SR-B-Patienten einen signifikant niedrigeren ISS aufwiesen als jene Patienten, die in gesamter Stärke des Schockraumteams angenommen wurden (ISS SR-A: 28,3 ± 21,5 vs. SR-B: 6,8 ± 7,9; *p* < 0,001; Abb. 3).

Im Rahmen der Patientenannahme und -versorgung erfolgte unabhängig von der Schockraumzuordnung auch eine Blutentnahme. Passend zum insgesamt schwereren Verletzungsmuster ist der Hämoglobinwert der SR-A-Patienten signifikant reduziert, wohingegen das Lactat signifikant erhöht ist (Tab. 3).

**Table 3:** 

Parameter	SR‑A	SR‑B	*p*-Wert
Hb (g/dl)	12,7 ± 2,5	14,1 ± 1,9	0,0099
Lactat (mmol/l)	3,05 ± 3,1	1,84 ± 1,2	0,0027
Base Excess (mmol/l)	1,25 ± 5,1	0,69 ± 2,6	0,0846
Thrombozyten (10^9^/l)	214 ± 82	235 ± 63	0,1175

Wertebereich angegeben als Mittelwert mit Standardabweichung

*Hb* Hämoglobin

### Interventionen im Schockraum

In 4 von 93 (4,3 %) SR-B-Versorgungen und bei 1 von 42 (2,4 %) SR-A-Patienten konnte in der Sonographie (eFAST) ein traumaassoziierter pathologischer Befund festgestellt werden. Die Durchführung des eFAST wurde in den SR‑A durch den anwesenden Facharzt für Viszeralchirurgie und in den SR‑B durch den Oberarzt der Notaufnahme durchgeführt. Um eine Untersucherabhängigkeit soweit wie möglich zu vermeiden, erfolgte vor Beginn dieser Studie eine interne Schulung.

Bei einem SR-A-Patienten musste anhand der klinischen und sonographischen Befunde eine Thoraxdrainage angelegt werden. In keinem der SR-B-Fälle war eine unmittelbare Intervention erforderlich.

### Bildgebung

Aufgrund der schwereren Verletzungen der SR-A-Patienten wurden in dieser Gruppe deutlich mehr Ganzkörper-Computertomographien („Polytraumaspirale“) durchgeführt (Tab. 4). Die kraniale Computertomographie (CCT) hingegen war in beiden Gruppen mit 80,9 % (SR-A) und 75,2 % (SR-B) relativ häufig erfolgt, wobei die im Rahmen der Polytraumaspirale durchgeführte CCT-Bildgebung miteingerechnet wurde. Ein konventionelles Röntgen wurde nur bei expliziter Fragestellung im Anschluss an den Primary Survey durchgeführt. Von regelhaften Röntgenübersichtsaufnahmen des Thorax und des Beckens wurde abgesehen und dies nur bei entsprechendem klinischem Verdacht resp. pathologischen Auffälligkeiten im eFAST ergänzt.

**Table 4:** 

Diagnostikum	SR‑A	SR‑B
CCT	80,9 % (*n* = 34)	75,2 % (*n* = 70)
Polytraumaspirale	58,1 % (*n* = 25)	44,1 % (*n* = 41)
Konventionelles Röntgen	45,2 % (*n* = 19)	59,1 % (*n* = 55)

### Intensivaufenthalt und Letalität

Im Studienzeitraum verstarben 2 Patienten (SR-A) noch während der Schockraumversorgung. Von den 14 intensivmedizinisch weiterbehandelten Patienten sind 6 (SR-A: *n* = 5; SR-B: *n* = 1) während des Intensivaufenthaltes verstorben; zwei weitere Patienten (SR-A) verstarben in tabula während der notfallmäßigen Operation.

Alle nichtintensivpflichtigen Schockraumpatienten wurden der klinikeigenen SOP „Schwerverletztenversorgung“ folgend, im Anschluss an die Schockraumversorgung für 4–6 h in der INA-Aufnahmestation engmaschig überwacht, erhielten eine zweite eFAST-Sonographie, wenn zuvor keine CT-Polytraumaspirale durchgeführt wurde, sowie eine Blutgasanalyse im Verlauf. In keinem der Fälle wurde eine vital bedrohliche Auffälligkeit festgestellt, welche eine Verlegung auf die Intensivstation oder eine interventionelle resp. operative Therapie erforderte. Von den SR-B-Patienten konnten schließlich 6 Patienten (6,5 %) bereits am Tag der Krankenhausaufnahme in die ambulante Versorgung entlassen werden.

## Diskussion

In unterschiedlichen Bundesländern und Regionen wird eine evidenz- und konsensbasierte Unterscheidung von A‑ und B‑Schockräumen vorgenommen. In der Regel wird eine Zuordnung bereits präklinisch vorgenommen.

Die Ergebnisse der prospektiven Studie demonstrieren ein sicheres Vorgehen bei der innerklinischen Schockraumalarmierung durch einen zuständigen Facharzt der Notaufnahme. Hierzu wurde eine Kombination aus den gemeldeten Informationen nach den aktuellen S3-Leitlinien Polytrauma und dem allseits angewendeten ABCDE-Schema genutzt. In keinem der Fälle musste bei einem SR‑B eine invasive Intervention durchgeführt werden oder das in Bereitschaft stehende SR‑A Team nachträglich hinzugezogen werden. Die Intensivüberwachungsrate war mit 3,2 % der SR-B-Patienten äußerst gering. In der SR-B-Gruppe ist darüber hinaus nur ein Patient im Rahmen des stationären Aufenthalts verstorben. Für diesen Patienten lag eine Patientenverfügung vor, nach der die Therapie entsprechend einzustellen war. Bei keinem anderen Patienten, der für den SR‑B triagiert wurde, wurde eine lebensgefährliche Diagnose gestellt. Passend dazu ist der ISS der SR-A-Patienten deutlich höher als bei den SR-B-Patienten.

Das Ziel aktueller Untersuchungen ist die Bewertung der Versorgungsqualität und Kosteneffizienz infolge dieser modifizierten Einteilung, da diese unterschiedliche personelle und instrumentelle Vorhaltekosten vorsieht und einen wesentlichen Einfluss auf klinikinterne Prozesse nimmt. Erste Studien haben gezeigt, dass sich durch die selektive Alarmierung von A‑ und B‑Schockräumen eine deutliche Ressourceneinsparung ergeben kann, ohne dass hierbei die Patientensicherheit gefährdet wird.

Sowohl Lerner et al. 2011 als auch Stephan et al. 2020 konnten zeigen, dass der Unfallmechanismus keine adäquate Vorhersage über die Verletzungsschwere ermöglicht und daher eine häufige Übertriage und somit inadäquate Inanspruchnahme von Ressourcen im Schockraum stattfindet. Es wird daher seit Jahren an der Optimierung der Kriterien zur Schockraumalarmierung gearbeitet, u. a. auch im Rahmen einer prospektiven Studie des TraumaRegister DGU® [4, 6].

In dem beobachteten Studienzeitraum fand nur in 86 Fällen (63,7 %) eine präklinische Notarztbegleitung statt. Vor diesem Hintergrund erscheint es fraglich, inwieweit die umfangreiche Schulung eines festgelegten präklinischen Algorithmus mit Leitdiagnosen, wie von Speringer et al. gefordert, mehr Sicherheit schafft und für die praktische Anwendung unter Berücksichtigung eines ressourcenschonenden Vorgehens sinnvoll ist. Weder den Mitarbeitern des Rettungsdienstes noch dem Notarzt kann trotz Vorgaben durch die S3-Leitlinie Polytrauma und dem Weißbuch der DGU zugemutet werden, die unterschiedlichen und krankenhausspezifischen Schockraumteamzusammensetzungen zu kennen. Im Gegensatz dazu können aber mit Hilfe des universellen ABCDE-Schemas und des Verletzungsmusters resp. des Unfallmechanismus Informationen an die Leitstelle weitergegeben werden, anhand derer dann eine innerklinische Zuordnung möglich ist. Es konnte in dieser Studie gezeigt werden, dass so die Balance zwischen innerklinischer Ressourcenvorhaltung und Patientensicherheit durch eine adäquate Schockraumzuordnung gewährleistet werden kann. Eine Folge dieses Vorgehens ist sicher auch eine Entlastung des präklinisch versorgenden Einsatzteams, da diese nur noch die Daten melden muss und die entsprechende Zuordnung in der Zielklinik stattfindet.

## Limitationen

Nachdem die Studie aufgrund der COVID-19 Pandemie verzögert gestartet wurde und die verschiedenen Infektionswellen die Notaufnahmen und das Personal im klinischen Alltag belasteten sowie das Alltagsverhalten der Menschen (z. B. Homeoffice, Einschränkungen in der Sportausübung) ganz entscheidend veränderten [8], sind der kurze Beobachtungszeitraum, die geringe Fallzahl sowie das monozentrische Design in ihrer Aussagekraft limitierend.

Im Studienzeitraum ist im Rettungsdienstbereich des Universitätsklinikums nur eine telefonische Schockraumanmeldung über die Leitstelle etabliert. Ein Behandlungskapazitätsnachweis oder ein digitales Zuweisungssystem mit Datenübermittlung aus den Rettungsmitteln ist noch nicht umgesetzt, was – wie auch in der Studie von Hagenbusch beschrieben [7] – zu weiteren Schwierigkeiten in der Schockraumanmeldung führen kann.

Eine adäquate Regelung außerhalb der Anwesenheit der Fachärzte in der INA stellt eine Herausforderung für das gesamte Team dar. Eben diese Phase wurde in dieser Studie jedoch nicht beleuchtet.

## Ausblick

Zum Zeitpunkt der Studie gibt es nach den Erkenntnissen der Autoren keine einzige nationale Studie über die innerklinische Zuordnung der Schockräume durch einen Facharzt der Notaufnahme anhand der Kombination aus ABCDE-Schema und dem Unfall‑/Verletzungsmuster der Leitstellenanmeldung.

Sowohl die Tatsache, dass bei 45 % der SR-B-Einweisungen kein Notarzt anwesend war, als auch die Studie von Hagenbusch et al. [7], die eine sehr heterogene Alarmierung des Schockraums aufweist, verdeutlichen den Nutzen weiterer klinikinterner „First-View-Konzepte“ in der Patientenzuordnung, wie in dieser Studie aufgezeigt. Auf diese Weise kann gerade bei den SR-B-Patienten eine zügige, ressourcenschonende und sichere Versorgung ermöglicht werden, ohne in die Gefahr einer Untertriage zu geraten.

Weitere zukunftsorientierte Konzepte sind erforderlich, da gerade in der erweiterten und umfassenden Notfallversorgung durch die Weiterentwicklung der Notaufnahmen die Chance zur adaptierten Schockraumaktivierung und Ressourcenmobilisierung im interdisziplinären Rahmen ermöglicht wird. Dabei werden neue Konzepte der Notaufnahmen so aufgestellt werden müssen, dass in Zukunft die Ressourcen der traumatologischen und der nicht-traumatologischen Schockräume sinnvoll und effizient rund um die Uhr durch die interdisziplinäre Notaufnahme (INA) koordiniert und behandelt werden.

### Infobox Schockraumteam: Mitglieder und Aufgaben

*Basisteam im traumatologischen Schockraum (SR-B)*

- Unfallchirurgie: Facharzt/-ärztin/Facharztstandard (Traumaleitung) + unfallchirurgischer Assistenzarzt/-ärztin
- Notaufnahme: INA-Facharzt/-ärztin (Schockraumleitung) + 2 INA-Fachpflegekräfte
- Radiologie/Neuroradiologie: Assistenzarzt/-ärztin + 1 MTRA
- Weitere Fachdisziplinen nach Meldebild

*Erweitertes Schockraumteam (SR-A)*

- Unfallchirurgie: Oberarzt/-ärztin + Facharzt/-ärztin (Traumaleitung) + unfallchirurgischer Assistenzarzt/-ärztin
- Notaufnahme: INA-Facharzt/-ärztin (Schockraumleitung) + 2 INA-Fachpflegekräfte
- Anästhesiologie: Oberarzt/-ärztin + Facharzt/-ärztin + 1 Anästhesiefachpflegekraft
- Allgemein- und Thoraxchirurgie: Facharzt/-ärztin (eFAST)
- Neurochirurgie: Oberarzt/-ärztin + Assistenzarzt/-ärztin
- Radiologie/Neuroradiologie: Facharzt/-ärztin + Assistenzarzt/-ärztin + 1 MTRA
- Weitere Fachdisziplinen nach Meldebild
